# Experimental test of genuine multipartite nonlocality under the no-signalling principle

**DOI:** 10.1038/srep39327

**Published:** 2016-12-20

**Authors:** Chao Zhang, Cheng-Jie Zhang, Yun-Feng Huang, Zhi-Bo Hou, Bi-Heng Liu, Chuan-Feng Li, Guang-Can Guo

**Affiliations:** 1 Key Laboratory of Quantum Information, University of Science and Technology of China, CAS, Hefei, 230026, China; 2 Synergetic Innovation Center of Quantum Information and Quantum Physics, University of Science and Technology of China, Hefei, 230026, P.R. China; 3 College of Physics, Optoelectronics and Energy, Soochow University, Suzhou, 215006, China; 4 Centre for Quantum Technologies, National University of Singapore, 3 Science Drive 2, Singapore 117543, Singapore

## Abstract

Genuine multipartite nonlocality (GMN) has been recognized as the strongest form of multipartite quantum correlation. However, there exist states that cannot violate the Svetlichny inequality derived from the standard definition of GMN, even though they possess GMN properties. The reason is that the standard definition of GMN allows correlations that permit signalling among parties, which is inconsistent with an operational definition. Here, for the first time, we present an experimental test of GMN in the no-signalling scenario, with a three-photon pure state |*ψ*_*s*_〉 and a noisy W state. The experimental results show that these states cannot violate the Svetlichny inequality. However, our results also demonstrate that they do violate a new inequality derived from the definition of GMN based on the no-signalling principle, i.e., these states can exhibit GMN under the requirement of no-signalling. Our results will be useful for the study and applications of GMN in quantum communications and quantum computation.

Quantum theory troubled many prominent physicists in the early twentieth century, including Albert Einstein. In 1935, Einstein and his colleagues Boris Podolsky and Nathan Rosen designed a thought experiment to demonstrate that quantum theory was incomplete^1^ and should be replaced by a more complete theory using local hidden variables. Almost thirty years later, Bell proved his famous theorem^2^,^3^, which states that no physical theory of local hidden variables can ever reproduce all of the predictions of quantum mechanics, by showing that the predictions of quantum theory for some bipartite quantum states are incompatible with those of deterministic local hidden-variable models and that these quantum states are therefore nonlocal. Quantum nonlocality offers advantages in quantum information tasks^3^, such as those related to quantum cryptography^4^,^5^, communication complexity^6^, randomness generation^7^, and measurement-based quantum computation^8^.

In studies of bipartite quantum nonlocality, Bells nonlocality was thoroughly investigated in the early years^9^,^10^,^11^,^12^,^13^,^14^,^15^,^16^,^17^. In recent years, the concept of quantum steering^18^, which refers to the asymmetric nonlocal correlations between the two subsystems, has attracted increasing attentions^19^,^20^, because of its unique asymmetric features and possible applications in one-way quantum key distribution^21^ and quantum subchannel discrimination^22^.

Compared with the bipartite case, multipartite quantum nonlocality has a much richer and more complex structure, because of the wide variety of classes of hierarchical nonlocality in multipartite systems. Among all forms of hierarchical multipartite nonlocality, genuine multipartite nonlocality (GMN) is the strongest. The notion of genuine multipartite nonlocality was first introduced and studied by Svetlichny in 1987, and he derived a Bell-type inequality, i.e., the Svetlichny inequality, for testing genuine tripartite nonlocality^23^. Furthermore, the tripartite notion of Svetlichny inequality has since been generalized to arbitrary *N*-partite system^24^ and higher-dimensional multipartite system^25^. Moreover, several experiments have been conducted^26^,^27^,^28^ to test GMN. However, it has recently been shown^29^ that according to Svetlichny’s definition, genuine N-partite nonlocality can be established by *m (m* ≤ (*N* − 1)) collaborating parties through local operations and classical communications, which is inconsistent with an operational framework for GMN. Thus, a new definition of genuine multipartite nonlocality under the no-signalling principle has been proposed^29^,^30^,^31^,^32^,^33^,^34^, since allowing signalling is incongruous with a physical perspective.

Besides the consideration of physical congruousness, the definition of GMN based on no-signalling principle would also benefit the study of quantum information processing in a practical way. One example is that, with the help of such definition, completely connected graph states are shown to be genuine multipartite nonlocal^31^. Other examples refer to device-independent multipartite quantum information processing protocols, such as communication complexity problems^6^, device-independent quantum cryptography^4^, randomness expansion^7^, etc. To be concrete, lets consider the device-independent quantum secret sharing protocol^35^: In this protocol, the multipartite state used is required to exhibit GMN for the device-independent purpose, even when noises are unavoidable in practical environment. Thus, one would prefer using the no-signaling definition of GMN when checking the GMN property of the employed multipartite state, because comparing with the Svetlichny definition, this definition will enable wider range of noisy states to be demonstrated as genuine multipartite nonlocal, which will be shown in our experiment.

In this paper, we experimentally prepare a specially designed three-photon pure state and a noisy W state, and reconstruct their density matrices via quantum state tomography. Numerical calculation with these density matrices shows that these two states can not violate the Svetlichny inequality. However, experimental testing of these states using the new inequalities derived for detecting genuine multipartite nonlocality under the no-signalling principle reveals obvious violations, i.e., these states can still exhibit GMN although they cannot violate the Svetlichny inequality. Such contrasting experimental results clearly reveal the advantage of the no-signalling-based definition for detecting GMN.

## Results

### Theory of genuine multipartite nonlocality under the no-signalling principle

Consider a multipartite system of n parties, which are labelled by the index set 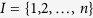. Each subsystem is measured by one observer. For the *k*-th subsystem (*k* ∈ *I*), the measurement setting and the outcome are denoted by *M*_*k*_ and *r*_*k*_, respectively. The joint outcome probabilities can be written in the form *P*(*r*_*I*_|*M*_*I*_), where 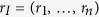 and 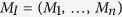. In a standard local hidden variable model, the (weakest form of) nonlocality implies that the joint outcome probability cannot be written as 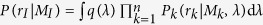, where *λ* is a shared local hidden variable, ∫*q*(*λ*)d*λ* = 1 for *q*(*λ*) ≥ 0, and *P*_*k*_(*r*_*k*_|*M*_*k*_, *λ*) is the probability of the *k*-th observer measuring observable *M*_*k*_ with outcome *r*_*k*_ for a given local hidden variable *λ*. By contrast, the GMN introduced by Svetlichny implies that the joint outcome probability cannot be written as


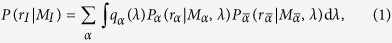


where *α* ≠ ∅, *α* ⊂ *I*, 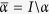, and 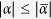. We divide the set *I* into two arbitrary nonempty subsets *α* and 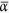. For every proper nonempty subset 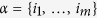, we denote its measurement settings by 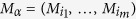 and its outcomes by 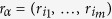, and *P*_*α*_(*r*_*α*_|*M*_*α*_, *λ*) denotes the joint probability of all observers *k* ∈ *α* measuring observable *M*_*k*_ with outcome *r*_*k*_ for a given local hidden variable *λ*. To discuss GMN in the no-signalling scenario, the no-signalling condition should be satisfied by all *P*_*α*_(*r*_*α*_|*M*_*α*_, *λ*) and 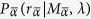 in Eq. (1), i.e.,


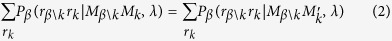


for all *k* ∈ *β, β* = *α* or 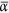, and |*β*| ≥ 2.

### Experimental scheme and results

To illustrate the difference between the Svetlichny inequality and the new inequalities derived based on the no-signalling principle^34^, we experimentally tested two cases: one was a special three-photon pure state 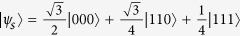, as proposed in ref. 34, and the other was a noisy W state.

**Figure 1:**
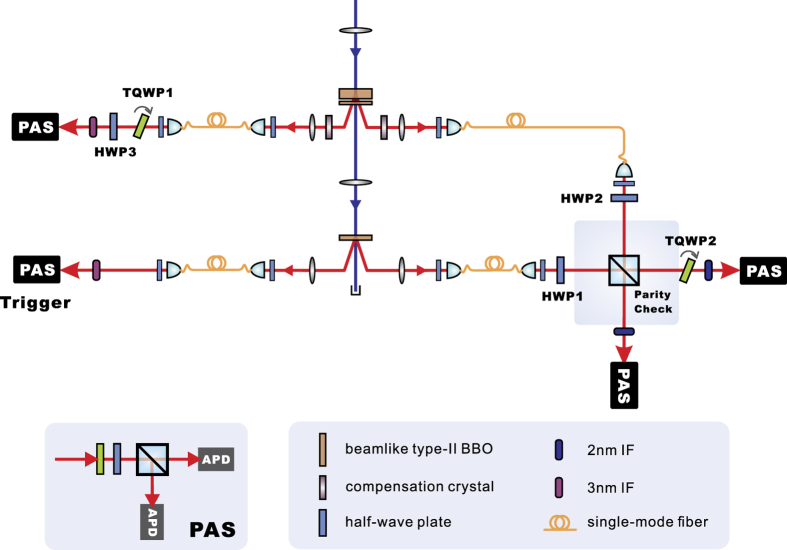
Experimental setup for the preparation and test of the tripartite pure state |*ψ*_*s*_〉. A femtosecond pulsed mode-locked Ti:Sapphire laser (with a central wavelength of 780 nm, a pulse duration of 90 fs and a repetition rate of 76 MHz) first passes through a frequency doubler. The emitted ultraviolet pulses are then used to pump two type-II SPDC sources. The first SPDC source produces a non-maximally entangled state. The compensation crystals are used to compensate for the spatial and temporal differences between the two orthogonal polarizations. The second source produces a product state. Single-mode fibers are used to further spatially filter the down-converted photons to be used as source outputs. Three half-wave plates (HWP1, HWP2, and HWP3) are placed at angles of (15°, 65.7°, −37.5°) to impose single-qubit rotations. Two extraordinary photons then interfere on a polarizing beam splitter (PBS) for a parity check operation. When there is one and only one photon in each of the spatial modes 1, 2, 3, and 4, the target state is generated via post-selection. Finally, each photon is measured by a polarization analysing system (PAS), which consists of one HWP, one quarter-wave plate (QWP), one PBS and two single photon detectors. The three terms of the target state have two independent relative phases, which can be tuned by two tiltable quarter-wave plates (TQWP1 and TQWP2).

For the pure state |*ψ*_*s*_〉, we find that it belongs to the Greenberger-Horne-Zeilinger (GHZ) class, and that it can be generated similarly to the standard GHZ state. Thus, it can be prepared by starting from the product of a single-photon state 
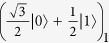
 (where |0〉 and |1〉 represent the |*H*〉 and |*V*〉 polarization states, respectively) and a two-photon entangled state 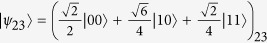 and then applying a parity check gate between photons 1 and 2, which can be achieved by causing them to interfere on a polarizing beam splitter (PBS). Through Schmidt decomposition, |*ψ*_23_〉 can be rewritten in terms of two orthogonal bases as follows: 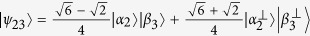, where 
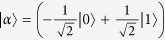
, 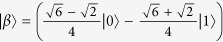, and the superscript ⊥ denotes the orthogonal state. This state is equal to 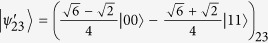, through several single-qubit rotations. The ratio between the modular square of the two coefficients for |00〉 and |11〉 is approximately 14:1. We have optimized the polarization direction of photon 1 to minimize this ratio. The experimental setup for the |*ψ*_*s*_〉 case is shown in Fig. 1. In the experiment, we used two spontaneous parametric down-conversion (SPDC) sources with beamlike phase-matching^36^,^37^ type-II *β*-barium borate (BBO) crystals to prepare the single-photon and two-photon states. To prepare the state 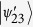, we used a sandwich-like source of entangled photon pairs similar to that in ref. 38. The only differences were that the thicknesses of the two BBO crystals here were 0.6 mm and 2 mm, respectively, and the temporal and spatial compensation crystals were a 6.58-mm quartz plate and a 3.2-mm LiNbO3 crystal, respectively, for extraordinary photons and a 8.43-mm quartz plate with no spatial compensation for ordinary photons.

**Figure 2:**
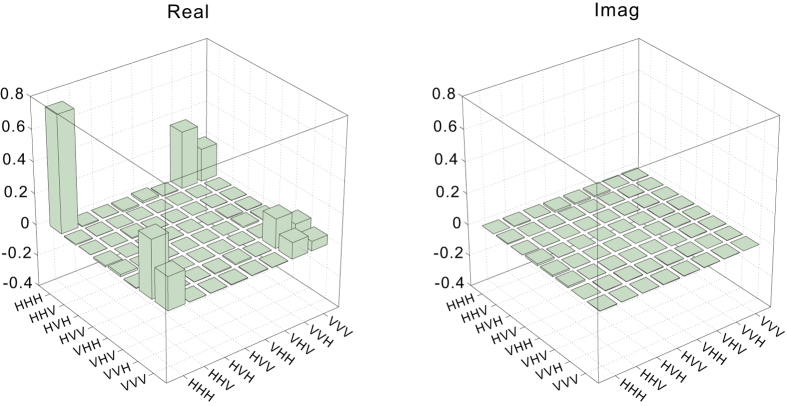
The real (left) and imaginary (right) parts of the density matrix 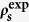 of the generated tripartite state |*ψ*_*s*_〉. The largest element of the imaginary part is less than 0.015.

We characterized the prepared state via quantum state tomography. The experimental data were obtained through measurements corresponding to 27 joint measurement settings, which included all possible combinations of the three Pauli operators for each qubit. To prepare |*ψ*_*s*_〉 with high fidelity, it is important to suppress the noise from the higher-order emission of photon pairs; thus, we used a pump power of only 15 mW, and we obtained a fourfold coincidence counting rate of 0.27/s. The total duration of the tomography measurements was 18 hours. We used the maximum likelihood approach to reconstruct the density matrix 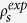. The result was well consistent with the target state, which can be quantitatively characterized by the state fidelity 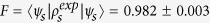. The real and imaginary parts of 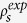 are shown in Fig. 2 (see Supplementary Information B for the raw tomography data). All of our experimental data have been corrected for the different detection efficiencies of the two single-photon detectors in each polarization analyzing system (PAS).

Using the reconstructed density matrix 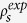, we can numerically optimize the measurement settings in the Svetlichny inequality to achieve its maximal possible value (calculated via quantum mechanics). The Svetlichny inequality is written as follows:


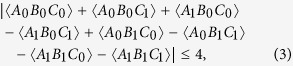


where A, B, C denote the three particles, and the subscripts 0, 1 denote two different measurements. With the optimized measurement settings (see Supplementary Information A), the maximal value of the left-hand side of the above inequality is calculated to be 3.77 ± 0.03, which is certainly less than 4. Thus, it is clear that our prepared state 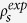 cannot violate the Svetlichny inequality.

Therefore, to detect the genuine tripartite nonlocality of 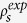, we can try to directly test a new inequality based on the no-signalling principle in experiment. We chose the class 16 inequality presented in ref. 30, which is rather robust against noises. This inequality is given below:


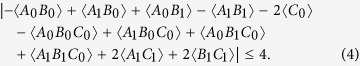


The expectation values can be calculated as 〈*A*_*X*_〉 = ∑_*a*_(−1)^*a*^*P*(*a*|*X*), 〈*A*_*X*_*B*_*Y*_〉 = ∑_*ab*_(−1)^(*a*+*b*)^*P*(*ab*|*XY*), etc., where *a, b* ∈ {0, 1} denote the binary outputs of the measurements. To test this inequality, we optimized the projection measurement basis (see Supplementary Information A) by numerical search (corresponding to |*ψ*_*s*_〉〈*ψ*_*s*_| instead of 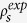) and then measured each photon in these basis with the PAS system, which used motorized wave plates to define the projection state. The motorized rotations of the wave plates had a high absolute accuracy of less than ± 0.05° and a good repeatability of less than 0.05°, which is important in such ‘black-box’ experiments. Our experimental result for this inequality was 4.34 ± 0.04, which is very close to the ideal value of 4.37 predicted by quantum mechanics and violates the inequality by 8 standard deviations. Note that the typical loopholes in Bell-type test are not closed here.

For mixed states, it is easier to find such states that cannot violate the Svetlichny inequality but can exhibit GMN in the no-signalling scenario. Similar to genuine multipartite entanglement, there is a threshold fidelity for noisy states to exhibit genuine multipartite nonlocality. Here we use the term “fidelity” to denote the fidelity between the ideal state and the noisy state. As an example, we experimentally generated a noisy three-photon W state and tested it using the inequality based on the no-signalling principle. The ideal W state without noise has the form of 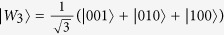. Similar to the case of the pure state, we also characterized the prepared state with state tomography and calculated its value for the Svetlichny inequality given the optimized measurement settings (see Supplementary Information A).

**Figure 3:**
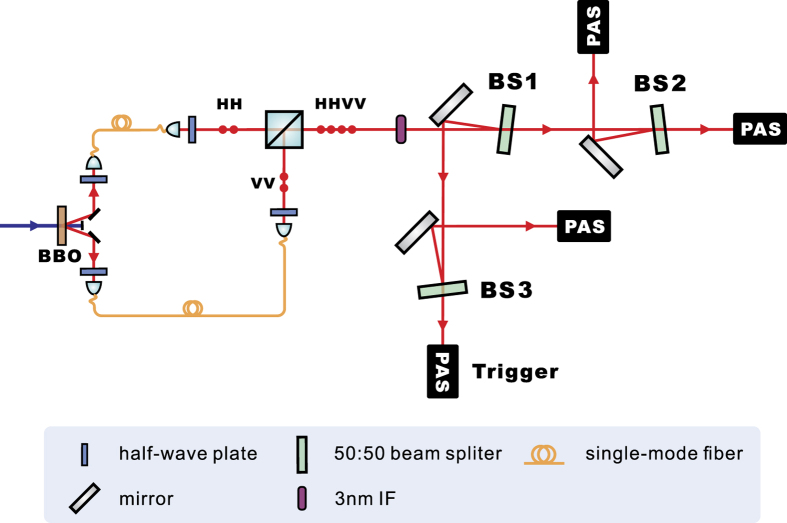
Experimental setup used to prepare the noisy three-qubit W state. The success probability of detecting one and only one photon in each spatial mode is 3/32. Each photon is ultimately measured by a PAS.

To generate the W state, we first prepared a four-photon Dicke state with two excitations, 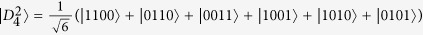. By projecting one photon into the state |*V*〉, the other three photons were prepared in the state |*W*_3_〉^39^. The experimental setup is shown in Fig. 3. We used two photon pairs generated in the second-order emission from one beamlike type-II SPDC source, and merged them into a single spatial mode with a PBS. The four indistinguishable photons were then randomly distributed into four different spatial modes by three 50:50 beam splitters (BSs). The superposition of the six possible distributions formed the state 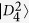. The four photons were spectrally filtered using interference filters (IFs) with bandwidths of 3 nm. The fidelity with which 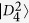 was prepared was measured to be 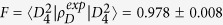. The density matrix 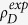 was obtained via quantum state tomography with a pump power of 35 mW and a four-photon coincidence rate of 0.1 Hz.

**Figure 4:**
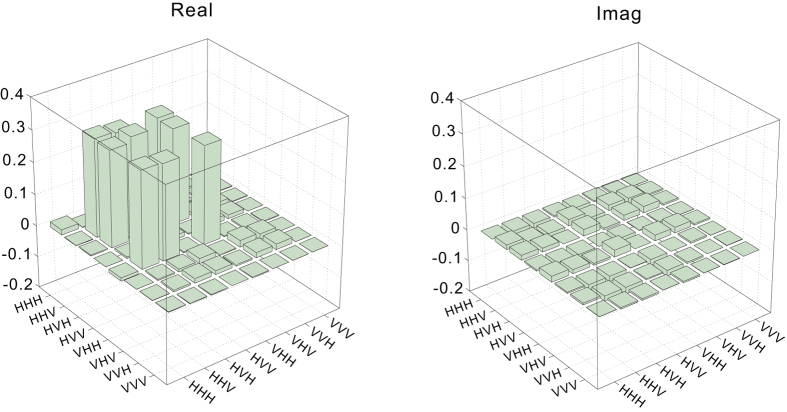
The real (left) and imaginary (right) parts of the reconstructed density matrix 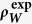 of the noisy W state.

A useful advantage of the new inequality based on the no-signalling definition is that it has stronger ability in finding GMN than the Svetlichny inequality. In particular, considering of the noisy W state, when its fidelity with the ideal W state is lower than a certain threshold, it cannot exhibit GMN using the Svetlichny inequality. However, using the no-signalling inequality, we still can demonstrate its GMN. To show such advantage, it is necessary to prepare a noisy W state with suitable fidelity. The method we used to add noise was simply to increase the pump power, thereby increasing the noise from higher-order photon pair emission, i.e., the noise caused by the emission of more than two photon pairs in our SPDC system. When the pump power was 210 mW, the fidelity was measured via quantum state tomography to be 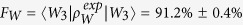, and the four-photon coincidence rate was 2.1 Hz after projection. Figure 4 shows the reconstructed density matrix 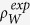 of the noisy W state (see Supplementary Information B for the raw tomography data). From 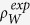, the maximal value of the Svetlichny inequality can be calculated to be 3.85 ± 0.04, which obviously shows no violation. It is worth to note that, although the noise introduced by high pump power is typically not a white noise, but a colored noise, the conclusion that this noisy W state cannot violate the Svetlichny inequality will not be affected. The reason is that the maximal Svetlichny inequality value obtained here was evaluated with numerically optimized measurement settings according to the measured density matrix 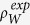.

Then we chose to test the following new inequality (labelled as class 138 in ref. 30):


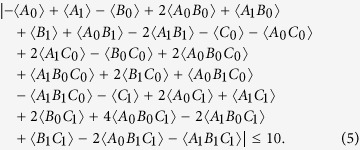


Again, with the numerically optimized measurement settings (see Supplementary Information A) corresponding to 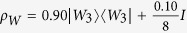 instead of 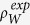, where *I* is a 8 × 8 identity matrix, we tested this inequality and obtained a value of 11.30 ± 0.07, which violates the bound of 10 by about 17 standard deviations. Note that, in the test of |*ψ*_*s*_〉 and here, we used an idealized state (|*ψ*_*s*_〉) or noise (the white noise *I*) as the target states for the numerical searches. The reason was to ensure that our experimentally prepared states can violate the inequalities, even in the absence of accurate or complete knowledge of their real density matrices. Moreover, because the experimental test of GMN is substantially a “black-box” experiment, when we experimentally obtain a violation, GMN is really demonstrated, even though the selected measurement settings are not optimal with respect to the practically generated state.

## Discussions

**Figure 5:**
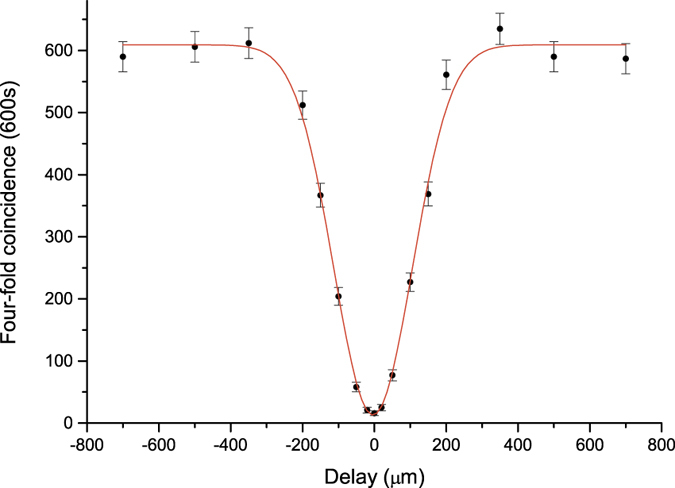
HOM interference fringe representing the HOM interference between different photon pairs measured with 2-nm and 3-nm IFs for e- and o-photons, respectively. The visibility is approximately 97.5%.

The dominant noise in our experiment was the temporal distinguishability between different photon pairs, which primarily depends on the ratio between the coherence length of the interfering down-converted photons and the pulse duration of the pump laser. We have finely adjusted the mode-locked Ti:Sapphire laser to minimize this parameter. It is done by finely tuning the position of the compensation prism in the laser to achieve a shorter output pulse width. We also use narrow-band spectral filters to increase the coherence length of the down-converted photons. In particular, in the experiment conducted to test the state |*ψ*_*s*_〉, state preparation with rather high purity was required for violation of the inequality. So we use 2-nm bandwidth Ifs for the extraordinary photons to achieve a higher visibility of interference, and 3-nm IFs for ordinary photons to achieve better efficiency photon pair collection. The pulse duration of the Ti:sapphire laser was measured to be 90 fs using an auto-correlator. With this measurement configuration, we observed the two-independent-photon Hong-Ou-Mandel (HOM) interference with a rather high visibility of 97.5% ± 0.6% (see Fig. 5), which to our knowledge is the highest two-independent-photon interference visibility ever reported.

In conclusion, we experimentally studied GMN in the no-signalling scenario with the |*ψ*_*s*_〉 state and the noisy W state. Our results clearly demonstrate that, although these states cannot violate the standard Svetlichny inequality, they can nevertheless violate the newly derived no-signalling-based inequalities, i.e., the multipartite correlations obtained from these states cannot be explained by any no-signalling local realism models. From the resource perspective, these results should be useful in achieving a substantial savings in multipartite nonlocal resources for various practical applications in multipartite quantum communication tasks. The results of this work are expected to be helpful for guiding the study and application of multipartite nonlocality in quantum information processing.

## Additional Information

**How to cite this article**: Zhang, C. *et al*. Experimental test of genuine multipartite nonlocality under the no-signaling principle. *Sci. Rep.* **6**, 39327; doi: 10.1038/srep39327 (2016).

**Publisher's note:** Springer Nature remains neutral with regard to jurisdictional claims in published maps and institutional affiliations.

## Supplementary Material

Supplementary Information
