# Supplementary material for: Experimental test of genuine multipartite nonlocality under the no-signalling principle
Source: Sci Rep. 2016 Dec 20;6:39327. doi: 10.1038/srep39327 (PMC5171240; doi:10.1038/srep39327)
Supplement: Supplementary Information [file srep39327-s1.pdf]

Supplementary Information: Experimental test of genuine multipartite nonlocality under no-signaling principle

Chao Zhang,[1,2] Chengjie Zhang,[3,4] Yun-Feng Huang,[1,2] Zhi-Bo Hou,[1,2] Bi-Heng Liu,[1,2] Chuan-Feng Li,[1,2] and Guang-Can Guo[1,2]

[1]*Key Laboratory of Quantum Information, University of Science and Technology of China, CAS, Hefei, 230026, China*
[2]*Synergetic Innovation Center of Quantum Information and Quantum Physics, University of Science and Technology of China, Hefei, 230026, P.R. China*
[3]*College of Physics, Optoelectronics and Energy, Soochow University, Suzhou, 215006, China*
[4]*Centre for Quantum Technologies, National University of Singapore, 3 Science Drive 2, Singapore 117543, Singapore*

(Dated: September 18, 2016)

A: Optimizing measurement settings

For a given multipartite nonlocality inequality and a known quantum state, the measurement operators (such as the A_0, B_0, C_0 and A_1, B_1, C_1 in the main text) should be properly chosen to achieve the maximal value of the inequality. For the Svetlichny and the new inequalities listed in the main text, we use a MATLAB program to numerically calculate the optimal measurement operators.

Rewriting the measurement operators A_i (or B_i, C_i) $(i = 0, 1)$ with projection operators as: $A_i = 2|a_i\rangle\langle a_i| - I$, where I is the identity operator and $|a_i\rangle = cos[\theta_i^a]|0\rangle + e^{i\phi_i^a} sin[\theta_i^a]|1\rangle$, we finally get the results listed in Table I and II.

TABLE I: Optimized measurement settings for the $|\psi_s$ state. All settings are in the unit of radians.

Settings	Svetlichny	No-signaling
θ_0^a	2.3464	1.1858
θ_0^b	0.8004	0.7664
θ_0^c	0.3474	0.5112
θ_1^a	2.3978	0.6763
θ_1^b	2.3358	1.2758
θ_1^c	2.8074	0.9620
ϕ_0^a	2.1527	0
ϕ_0^b	0.9827	0
ϕ_0^c	0.7826	0
ϕ_1^a	0.6038	0
ϕ_1^b	2.5308	0
ϕ_1^c	2.3458	0

B: Raw data of quantum state tomography.

We use the over-complete QST here. The experimental data is obtained by measuring 27 joint measurement settings which consist of all combinations of the three Pauli operators (Z, X, Y) for each qubit. Table III and IV list the raw four-photon coincidence counts recorded in the QST for the $|\psi_s\rangle$ state and the noisy W state, respectively. In the table, each line corresponds to a joint measurement setting, such as ZZZ, ZYX, etc. For each Pauli operator, there are two possible results: 1 or -1. So, each column in the table corresponds to one of the 8 possible results (such as 111, 1-11, etc.) in one joint measurement setting.

TABLE II: Optimized measurement settings for the noisy W state. All settings are in the unit of radians.

Settings	Svetlichny	No-signaling
θ_0^a	1.8986	0.6651
θ_0^b	1.8490	0.6651
θ_0^c	1.2387	3.0243
θ_1^a	1.2658	1.4535
θ_1^b	1.2247	1.4535
θ_1^c	0.3001	0.6651
ϕ_0^a	0.6769	0
ϕ_0^b	0.7121	0
ϕ_0^c	0.7741	0
ϕ_1^a	0.8834	0
ϕ_1^b	0.7891	0
ϕ_1^c	0.6358	0

TABLE III: Raw data for QST of the $|\psi_s\rangle$ state. The data collection time for each joint measurement setting is 2400 s.

Setting	111	11-1	1-11	1-1-1	-111	-11-1	-1-11	-1-1-1
ZZZ	491	1	4	1	0	0	114	32
ZZX	245	264	4	1	1	0	120	7
ZZY	228	217	2	3	0	2	90	78
ZXZ	229	0	250	1	72	15	54	20
ZXX	145	118	125	126	72	2	66	6
ZXY	139	159	118	104	43	40	52	57
ZYZ	250	0	235	3	57	26	60	30
ZYX	110	107	117	127	78	6	73	5
ZYY	131	110	116	127	30	32	47	51
XZZ	280	0	50	7	208	1	83	24
XZX	138	127	70	9	114	113	84	6
XZY	119	133	46	44	120	121	62	29
XXZ	270	15	37	9	21	17	253	11
XXX	201	96	3	44	4	40	210	70
XXY	129	147	24	19	28	20	137	147
XYZ	130	6	164	10	136	8	156	8
XYX	95	70	112	88	107	54	94	90
XYY	55	110	124	47	121	38	44	119
YZZ	268	0	72	17	236	1	67	23
YZX	116	106	76	9	113	122	79	7
YZY	132	111	35	52	119	124	44	49
YXZ	165	5	152	12	128	6	137	11
YXX	101	58	92	65	90	76	83	56
YXY	62	107	96	37	109	49	40	127
YYZ	48	8	292	7	260	14	29	18
YYX	7	29	174	83	203	88	0	50
YYY	15	19	151	150	149	160	22	20

TABLE IV: Raw data for QST of the noisy W state. The data collection time for each joint measurement setting is 640 s.

Setting	111	11-1	1-11	1-1-1	-111	-11-1	-1-11	-1-1-1
ZZZ	21	424	458	14	372	22	29	0
ZZX	222	230	202	225	218	241	7	12
ZZY	235	197	178	225	193	230	6	11
ZXZ	211	196	209	186	226	18	202	14
ZXX	435	20	17	406	133	102	108	129
ZXY	206	225	234	196	88	105	106	125
ZYZ	232	164	185	228	191	11	212	15
ZYX	226	204	203	249	96	112	150	123
ZYY	386	15	16	375	105	101	96	133
XZZ	193	230	225	11	236	248	192	14
XZX	390	9	145	122	19	404	99	110
XZY	189	205	113	136	213	259	94	132
XXZ	409	129	15	105	26	104	401	107
XXX	501	52	57	65	61	61	61	446
XXY	243	319	59	61	50	53	242	259
XYZ	203	99	228	131	208	98	217	123
XYX	278	67	298	63	68	251	56	264
XYY	248	76	52	298	266	55	54	261
YZZ	251	183	174	6	184	224	262	6
YZX	219	219	103	105	195	232	141	140
YZY	363	12	90	98	12	417	95	158
YXZ	258	108	190	88	200	133	237	103
YXX	279	63	57	284	312	66	59	284
YXY	259	67	232	47	70	287	51	295
YYZ	424	109	26	97	23	95	447	138
YYX	283	258	60	58	52	47	285	306
YYY	456	75	77	62	68	46	48	496
